# Oxidation of Innate Immune Checkpoint CD47 on Cancer Cells with Non-Thermal Plasma

**DOI:** 10.3390/cancers13030579

**Published:** 2021-02-02

**Authors:** Abraham Lin, Jamoliddin Razzokov, Hanne Verswyvel, Angela Privat-Maldonado, Joey De Backer, Maksudbek Yusupov, Edgar Cardenas De La Hoz, Peter Ponsaerts, Evelien Smits, Annemie Bogaerts

**Affiliations:** 1 PLASMANT-Research Group, University of Antwerp, 2610 Antwerpen-Wilrijk, Belgium; Jamoliddin.Razzokov@uantwerpen.be (J.R.); hanne.verswyvel@uantwerpen.be (H.V.); Angela.PrivatMaldonado@uantwerpen.be (A.P.-M.); maksudbek.yusupov@uantwerpen.be (M.Y.); annemie.bogaerts@uantwerpen.be (A.B.); 2 Center for Oncological Research (CORE), Integrated Personalized & Precision Oncology Network (IPPON), University of Antwerp, 2610 Antwerpen-Wilrijk, Belgium; evelien.smits@uza.be; 3 Tashkent Institute of Irrigation and Agricultural Mechanization Engineers, Tashkent 100000, Uzbekistan; 4 Department of Physics, National University of Uzbekistan, Tashkent 100174, Uzbekistan; 5 Department of Biomedical Sciences, University of Antwerp, 2610 Antwerpen-Wilrijk, Belgium; Joey.DeBacker@uantwerpen.be; 6 Optical Metrology, 3D Design and Mechanics (Op3Mech) Research Group, University of Antwerp, 2020 Antwerpen, Belgium; Edgar.Cardenas@uantwerpen.be; 7 Laboratory of Experimental Hematology, Vaccine and Infectious Disease Institute, University of Antwerp, 2610 Antwerpen-Wilrijk, Belgium; peter.ponsaerts@uantwerpen.be

**Keywords:** non-thermal plasma, dielectric barrier discharge, cancer therapy, immune checkpoint, CD47, molecular dynamic simulation, reactive oxygen species

## Abstract

**Simple Summary:**

Non-thermal plasma is being developed for cancer immunotherapy. The aim of our study was to determine the effect of non-thermal plasma on immunosuppressive immune checkpoint, CD47. The direct effect of non-thermal plasma on CD47 was measured in vitro and in vivo, and the mechanism of action was studied in silico. Non-thermal plasma immediately oxidized CD47, suggesting a dual role of non-thermal plasma therapy to simultaneously increase immunogenic signals and reduce immunosuppressive ones.

**Abstract:**

Non-thermal plasma (NTP) therapy has been emerging as a promising cancer treatment strategy, and recently, its ability to locally induce immunogenic cancer cell death is being unraveled. We hypothesized that the chemical species produced by NTP reduce immunosuppressive surface proteins and checkpoints that are overexpressed on cancerous cells. Here, 3D in vitro tumor models, an in vivo mouse model, and molecular dynamics simulations are used to investigate the effect of NTP on CD47, a key innate immune checkpoint. CD47 is immediately modulated after NTP treatment and simulations reveal the potential oxidized salt-bridges responsible for conformational changes. Umbrella sampling simulations of CD47 with its receptor, signal-regulatory protein alpha (SIRPα), demonstrate that the induced-conformational changes reduce its binding affinity. Taken together, this work provides new insight into fundamental, chemical NTP-cancer cell interaction mechanisms and a previously overlooked advantage of present NTP cancer therapy: reducing immunosuppressive signals on the surface of cancer cells.

## 1. Introduction

Non-thermal plasma (NTP), also referred to as cold atmospheric plasma, has been emerging as a therapeutic modality against cancer [1]. NTP, a partially ionized gas generated at atmospheric pressure and room temperature, is composed of several physical and chemical components (e.g., electric fields, charged particles, reactive chemical species) [2,3]. It is now well established that the anti-cancer capacity of NTP is derived from the reactive oxygen and nitrogen species (RONS) it generates, including hydrogen peroxide (H_2_O_2_), nitrite (NO_2_^−^), peroxynitrite (ONOO^−^), and other radical species. Reports of NTP inducing apoptosis and necrosis and inhibiting cancer cell growth, without adverse side effects, makes it attractive for cancer therapy.

In recent years, it was proposed that NTP has cancer immunotherapeutic properties via induction of immunogenic cell death (ICD). ICD is a form of cell death that renders tumor cells more detectable to immune cells through emission of damage-associated molecular patterns (DAMPs), such as surface calreticulin (CRT), released high mobility group box 1, and secreted adenosine triphosphate, and stimulates the anti-cancer immune response [4,5]. Preclinical studies have demonstrated the ability of NTP to induce emission of several DAMPs in vitro [6,7,8,9] and in vivo [9,10,11].

Our lab has previously established an ICD-inducing regime of NTP using a vaccination assay, currently considered the ‘gold standard’ for identifying ICD agents [8]. We determined that the chemical species responsible for eliciting this effect in that regime were short-lived RONS, particularly hydroxyl radials (•OH), atomic oxygen (O), ozone (O_3_), and nitric oxide (•NO), while the more persistent species, with lifetimes greater than a second (e.g., H_2_O_2_, NO_2_^−^, NO_3_^−^, and ONOO^−^), had no effect [8]. Since NTP-generated RONS would first interact with cell membranes during treatment, we hypothesized that they will also oxidize and destroy membrane-bound proteins. This is advantageous for cancer immunotherapy as immunosuppressive surface proteins and checkpoints are often overexpressed on cancerous cells.

Advances in cancer immunotherapy in the past decade have been spurred by research into inhibitory immune checkpoints [12]. CD47, a transmembrane protein overexpressed on malignant cells in several types of tumors, is a checkpoint of the innate immune system that has garnered attention [13,14,15]. Upon binding with signal-regulatory protein alpha (SIRPα), found on innate immune cells [16], activation of pro-phagocytic receptors is prevented, and subsequently phagocytosis is sequestered [17,18]. Thus, CD47 is often known as a ‘don’t eat me’ signal and contributes to immune evasion of cancerous cells [19]. Therefore, disruption of the CD47–SIRPα pathway is of great interest.

In this study, we investigated the potential of NTP, operated in our previously defined ICD-inducing regime, to alter surface CD47 on cancer cells. The effect of NTP on CD47-expressing cancer cells was identified in vitro and in vivo, and the underlying mechanism of action was investigated in silico. In vitro, three different cancer cell lines were exposed to NTP in 3D tumor models. CD47 was evaluated immediately and 24 h after treatment. Melanoma tumors, established subcutaneously in syngeneic mice, were also treated with NTP, resected, and evaluated for CD47. Molecular dynamics (MD) simulations were performed to determine the structural changes of CD47 after oxidation and its subsequent influence on its binding affinity with SIRPα. Our in vitro results revealed that CD47 was modulated immediately after NTP treatment. In our 3D model of melanoma, this effect persisted up to 24 h after treatment. NTP treatment not only reduced tumor size, but also caused a slight reduction in CD47. The MD simulations indicated that oxidation of CD47 induced conformational changes to the protein, disrupts the Lys39-Asp100 and Lys41-Asp100 salt bridges in the CD47–SIRPα system, and subsequently reduced the binding affinity. Taken together, our in silico results and validation assays complement each other in evidencing the ability of NTP treatment to oxidize immunosuppressive CD47 on cancerous cells, which may affect downstream binding to innate immune cells. This study provides crucial, fundamental insight into NTP effects on cancer cells, and potentially other immune checkpoints, while supporting its role for cancer immunotherapy.

## 2. Materials and Methods

### 2.1. Cell Culture

Human glioblastoma (U87), melanoma (A375), and head and neck squamous cell carcinoma (SC263) cell lines were used in this study. All cell lines were cultured in Dulbecco’s modified Eagle medium containing 10% fetal bovine serum (Gibco, Loughborough, United Kingdom) and 100 U/mL penicillin, 100 μg/mL streptomycin (Gibco). L-Glutamine was also supplemented in the media: 2 × 10^−3^ M for U87 and SC263 and 4 × 10^−3^ M for A375. Cells were cultured in a humidified environment with 5% CO_2_ at 37 °C before seeding for experiments.

### 2.2. 3D Spheroid Model

For spheroid culture, U87 and SC236 cells were seeded into specialized round-bottomed, hydrogel coated 96-well plates (ultra-low attachment plates, ULA, Corning^®^ 7007, Corning, Amsterdam, The Netherlands). Cell suspensions were prepared with a concentrations of 5 × 10^4^ cells/mL, supplemented with 2% Matrigel (8.6 mg/mL, Corning) to enhance spheroid formation. Cells were seeded at a concentration of 5000 cells/well in 100 µL of culture medium and centrifuged for 10 min at 1000 RPM. Spheroids were formed in three days of undisturbed incubation at 37 °C and 5% CO_2_.

### 2.3. In Ovo Model

Fertilized chicken eggs (4-day old) were incubated for 1 day at 37.7 °C and 65% humidity in an egg incubator with automatic turning function (Ova-Easy 100, Brinsea, Veenendaal, The Netherlands). On day 5, the upper pole was disinfected and pierced with a 20 G sterile needle (BD) and sealed with medical tape (Leukosilk S, Covamed Farma BVBA, Marke, Belgium) to promote the relocation of the air chamber to the upper pole. The eggs were incubated in vertical position (without turning) until day 7. The eggshell was cut to expose the CAM and a 1 × 1 mm filter paper soaked in diethyl ether (Fisher Scientific, Merelbeke, Belgium) was briefly applied on a vascularized region of the CAM. A sterile silicone ring (ID = 5 mm, OD = 6 mm, 1 mm thickness) was placed on the CAM and 2 × 10^6^ A375 cells mixed with 15 µL growth factor reduced Matrigel (8.6 mg/mL, Corning, Amsterdam, The Netherlands) were loaded into the ring. The eggs were sealed with Tegaderm (3D) and placed back in the incubator for four days. On day 11, the Tegaderm was cut and the tumors were treated with NTP. Tumors were excised immediately or 24 h after treatment for CD47 analysis. All steps outside the incubator were carried out using a heat block (Corning, Lasne, Belgium) set at 37.7 °C with a custom-made egg-shaped aluminum adapter.

### 2.4. NTP Treatment

A microsecond-pulsed DBD system was used to generate NTP for treatment of 3D spheroids and in ovo tumors (30 kV output, 1–1.5 µs rise time, and 2 µs pulse width). Right before NTP treatment of the spheroids, spheroids were transferred to an empty flat-bottomed 96-well plate in 3 µL of culture medium. The DBD electrode (3 mm diameter) was lowered into the well, using a z-axis positioner, 1–2 mm above the cells. The NTP pulse frequency and treatment time was fixed at 500 Hz and 10 s, respectively. Immediately after treatment, 150 µL of fresh complete medium was added to the well and the spheroids were either collected immediately or placed back into incubation until further analysis. The DBD electrode was held by hand for treatment of A375 tumors in the in ovo model. The treatment was fixed at one position for 10 s, and the pulse frequency was increased to 700 Hz, to account for the larger tumor size. This setting is similar to what we used previously in subcutaneous tumors in vivo [10]. The tumors were resected immediately for analysis or incubated further for 24 h before collection.

### 2.5. In Vivo Model and Treatment

Female C57BL/6J mice, 8-week old, were purchased from Charles River (Charles River Laboratories, Wilmington, MA, USA) and housed in a pathogen-free room at the Animal Center of the University of Antwerp. 10^6^ B16F10 melanoma cells were subcutaneously injected into the left flank of each mouse. NTP treatment was initiated when tumors became palpable (18.2 ± 4.8 mm^3^), 3 days after melanoma cell injection. The NTP device was held ~1 mm above the tumor and treatment was performed for 10 s at 700 Hz. Mice were treated once a day for 5 consecutive days. Tumors were collected immediately after the last treatment or 72 h after the last treatment for analysis. Two orthogonal diameters were measured on the tumor (length and width) using digital calipers and volumes were calculated using 0.5 × length × width^2^. All animal experiments were approved by the University of Antwerp Animal Research Ethical Committee (ECD-dossier 2017-53).

### 2.6. Flow Cytometry Analysis of CD47 on Cell Lines

CD47 was measured using dual staining of 7-aminoactinomycin D (7AAD), a viability stain, and a monoclonal CD47 antibody. Prior to staining, the cells were washed with PBS, detached with 200 µL of accutase, and washed twice with 2 mL of FACS buffer (500 mL sheath fluid (342003, BD Biosciences, Aalst, Belgium) + 2 g bovine serum albumin (A9418, Sigma-Aldrich, Overijse, Belgium) + 1 g NaN_3_ (1.06688.0100 Merck, Overijse, Belgium) in 100 mL H_2_O). The samples were stained with 10 µL of PE mouse anti-human CD47 (556046, BD Biosciences) or with 10 µL PE Mouse IgG1, κ isotype control (555749, BD Biosciences) for 40 min at 4 °C. The cells were then washed with FACS buffer and 2 µL of 7AAD (420403, Biolegend, London, United Kingdom) was added to each sample for 15 min before being quantified with a flow cytometer (CytoFLEX flow cytometer, Beckman Coulter, Indianapolis, IN, USA). 10,000 events were collected and only viable cells (7AAD−) cells were analyzed for CD47 (Figure S1). Data were analyzed and gated using the FlowJo software version 10 (FlowJo LLC, Ashland, OR, USA).

### 2.7. Immunofluorescence Analysis of CD47 on Spheroid and Tumor Sections

The spheroids were collected immediately and 24 h after treatment and fixed overnight in 4% paraformaldehyde at 4 °C. The fixed spheroids were transferred to microarray molds of 4% agarose as described before [20]. The solidified agarose microarray supports were dehydrated and embedded in paraffin overnight. In ovo tumors derived from the CAM assay were also collected immediately or 24 h after treatment and fixed with 4% paraformaldehyde for 14 h at 37 °C prior to paraffin embedding. Both sample types were taken together for the remaining part of the immunofluorescence staining protocol. Slides of 5 µm were cut, deparaffinised and rehydrated. Heat-induced antigen retrieval was performed with sodium citrate buffer (10 mM sodium citrate, 0.05% Tween 20, pH 6.0) at 96 °C for 20 min. Slides were washed with washing buffer (TBS plus 0.025% Triton X-100) with gentle agitation (2 × 5 min) before blocking in 10% bovine serum albumin in TBS at room temperature for 2 h. Incubation with mouse monoclonal anti-CD47 primary antibody was done overnight at 4 °C (1/40 dilution; clone B6H12.2, MA5-11895, ThermoFisher Scientific, Merelbeke, Belgium). All slides were washed with washing buffer (2 × 5 min) and subsequently stained with Alexa Fluor 594-conjugated Donkey anti-Mouse IgG (H + L) highly cross-adsorbed secondary antibody for CD47 (1/500 dilution, Cat. No. A-21203, ThermoFisher Scientific). Incubation with secondary antibody at room temperature for 1 h was performed in the dark to avoid photobleaching. Lastly, all slides were rinsed with washing buffer (3 × 5 min), mounted with VECTASHIELD^®^ Antifade mounting medium containing counter staining DAPI (H-1200, VECTOR Laboratories, Peterborough, United Kingdom). Images were taken with an Olympus BX51 fluorescence microscope (Cat. No. WS-BX51-0169, Olympus Life Sciences, Calcutta, India).

All slides within the same experiment were imaged on the same day, and all images in this study were taken at fixed microscope and capture settings. Images were also batch processed in ImageJ to limit additional variation and potential bias. CD47 expression was quantified by averaging (arithmetic mean) the ratio of mean fluorescence intensities (MFI) of CD47 and DAPI (MFI_CD47_/MFI_DAPI_). For the spheroids, the entire spheroid was analyzed. For the in ovo tumor sections, three representative areas (100 µm × 100 µm) with only human cells were analyzed (Figure S2). This ratio accounts for the influence of the number of cells under observation on CD47 intensity within that region. The final ratio was normalized to the mean of the untreated section.

For in vivo evaluation of CD47, the images were measured using by using DAPI to binary mask for the locations of the individual nuclei. This was accomplished by taking the raw fluorescent intensities for DAPI and removing background signal and luminance artifacts through the use of a one sided low pass filter formed by a Gaussian kernel of 25 pixels (10 µm) in standard deviation. A kernel size of 25 was used for 20× images and 12 for 10×. Resultant values where saturated to 95th percentile of the image intensities and binarized to 0.40 of the normalized dynamic range. Individual nuclei in the images were indexed using connected component analysis and objects smaller than 20 pixels^2^ (100 µm^2^) were removed. The masks of each of the nuclei were dilated using a circular structuring element with diameter of 51 pixels (20.4 µm^2^) to form a tissue mask. Each pixel of the resulting tissue mask was indexed to the nearest nuclei. To correct for background signal, images were taken with tissue and non-tissue areas, and fluorescence intensity of the CD47 channel was preprocessed by saturating on the lower side to 5th percentile of the raw image intensity. Finally, the mean fluorescence intensity for each cell of the CD47 channel was taken for each nuclei, by measuring the overlap with the nuclei mask, overlap with the tissue mask or both and the mean across all nuclei for each of these metrics was reported for per tissue.

### 2.8. Statistical Analysis

Statistical differences for the experimental studies were analyzed using the linear mixed model with JMP Pro 13 (SAS Software, Tervuren, Belgium). NTP treatment was set as the fixed effect and the random effects tested include the date the experiment was performed or when the slides were imaged. The interactions between treatment and the date were also tested. The random slope model was only used when the interaction was significant (*p* ≤ 0.05). The fixed effect test was used to determine if there was a statistical difference between treatment (*p* ≤ 0.05), and the Dunnett’s test for statistical significance was performed post-hoc to calculate the adjusted p value compared to untreated controls. A p value less than or equal to 0.05 was considered statistically significant. Data are represented as mean ± SEM and all individual values are reported.

### 2.9. Computational Details

We performed MD simulations in order to understand the interaction mechanisms of the native and oxidized CD47 with SIRPα and B6H12.2 antibodies at the molecular level. The GROMACS 5.1 software was employed for the simulations, applying the GROMOS 45a3 force field [21]. As model systems we used the CD47-SIRPα and CD47-B6H12.2 complex structures obtained from the Protein Data Bank (ID: 2JJT and 5TZU).

The preparation steps of the model systems are given in detail in the supplementary materials. Briefly, we used two model structures, i.e., the native CD47–SIRPα (or CD47-B6H12.2) and oxidized CD47_OX_–SIRPα (or CD47_OX_–B6H12.2) protein complexes. These systems were placed in simulation boxes filled with water molecules including 0.1 M Na^+^ and Cl^−^ ions. Prior to the equilibration, the systems were energy minimized using the steepest descent algorithm. Subsequently, a 50 ps equilibration run was carried out applying the NVT ensemble (i.e., a system with constant number of particles N, volume V, and temperature T) and position restraints on the heavy atoms of the proteins. Further, the model systems were equilibrated for another 50 ps applying weak coupling thermo- and barostats and employing NPT ensemble (i.e., a system with constant number of particles N, pressure p, and temperature T). Finally, 350 ns production runs were performed using again the NPT ensemble, but employing the Nose-Hoover thermostat and the isotropic Parrinello-Rahman barostat, in this case removing the applied position restraints. All simulations were carried out at 310 K and 1 bar, and 1.4 nm cut-off distance was used for the Coulomb and van der Waals interactions. The trajectory of the production runs was used for data collection, i.e., for calculation of the root mean square deviation (RMSD) and distance between salt bridges of the CD47/CD47_OX_ and SIRPα/B6H12.2 complexes. The PyMOL molecular graphics tool was used to prepare the images presented in this paper.

From the last 50 ns trajectory of each 350 ns production run, we extracted three systems (at 300, 325 and 350 ns) for both native and oxidized complexes (i.e., 3 × 4 = 12 model systems in total). These systems were further used in our umbrella sampling (US) simulations. In the US simulations, we used 30 windows separated by 0.1 nm, which were extracted from the pulling simulations of CD47/CD47_OX_. Note that SIRPα (or B6H12.2) was restrained and used as a reference in the pulling simulations, and the pulling of CD47/CD47_OX_ was performed along the *z*-axis. Moreover, the movement of CD47/CD47_OX_ in the xy-plane was also restrained by using the so-called flat-bottomed position restraint, with a radius of 0.1 nm and a force constant of 500 kJ/(mol nm^2^). A 1000 kJ/(mol nm^2^) spring constant together with 0.001 nm/ps pulling rate were applied in the pulling simulations, which led to the disintegration of CD47/CD47_OX_ from SIRPα (or B6H12.2). Using each individual umbrella window, we further performed US simulations for 25 ns and the last 23 ns trajectory was used for data collection, i.e., the initial 2 ns was used to relax the system. Finally, the weighted histogram analysis method (WHAM) [22] was employed to calculate the potential of mean forces or free energy profiles (FEPs) of the dissociation of CD47/CD47_OX_ from the SIRPα (or B6H12.2) protein complex. The final FEP for each complex was averaged from three FEPs. The errors associated with the FEPs were estimated by means of the bootstrapping method. In total 30 × 3 × 4 = 360 US simulations were performed to obtain the final FEPs.

## 3. Results

### 3.1. NTP Effect on CD47 Using 3D In Vitro Models

**Figure 1:**
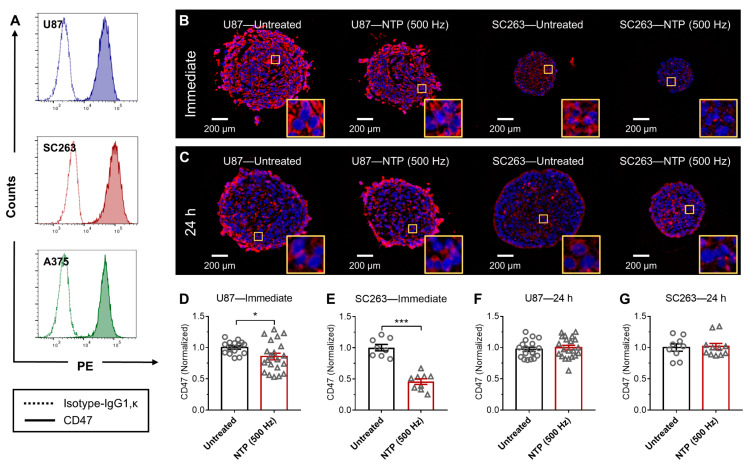
NTP treatment modulated CD47 immediately in 3D tumor spheroids. (**A**) The baseline expression of three human cancer cells, glioblastoma (U87), head and neck squamous cell carcinoma (SC263), and melanoma (A375) was analyzed using immunohistochemistry and flow cytometry. U87 and SC263 cells were able to form compact spheroids and were exposed to NTP. Spheroids were collected (**B**) immediately or (**C**) 24 h after NTP treatment, paraffin-fixed, sectioned, stained for CD47 (red), and counter-stained with a nuclear dye, 4′,6-diamidino-2-phenylindole (DAPI) (blue). Images were taken together at fixed microscope settings (10×) per experiment and all images were batch processed. Yellow inserts are a zoomed-in area (100 µm × 100 µm) to show CD47 staining surrounding the nucleus. CD47 expression was quantified and normalized to the untreated (**D**–**G**). Data here are represented as mean ± SEM and individual values are also shown (n = 8–21). Statistical significance of all treatment conditions was determined using the generalized linear mixed model with post hoc Dunnett’s test comparison to untreated (* *p* ≤ 0.05; *** *p* ≤ 0.001).

To assess the ability of NTP to modulate surface CD47 on cancerous cells, we used three different cancer cell types: glioblastoma (U87), head and neck cancer squamous cell carcinoma (SC263), and melanoma (A375). A screening of the baseline CD47 expression was first assessed with immunohistochemistry and flow cytometry analysis following dual staining with an anti-CD47 monoclonal antibody and a live-dead stain. Detailed gating strategies for analysis are provided in supplementary materials (Figure S1), and analysis of CD47 was only performed on live cell populations (7AAD^−^ population). When compared to their isotype controls (IgG1, κ), it was clear that all cell lines were 100% positive for CD47 (Figure 1A).

3D tumor spheroids were used to evaluate NTP effects on surface CD47, as spheroids better represent the pathophysiological development of cancer cells compared to 2D monolayers [23]. This model avoids changes in morphology, provides more realistic nutrient access and proliferation profile, and includes crucial features of the tumor microenvironment, which are absent in 2D monolayers [24]. Both the U87 and SC263 cell lines were able to form compact spheroids, but the A375 cell line was not, despite the use of different seeding concentrations and supportive matrices (Figure S3). This is likely due to the cancer type and phenotypic dependence of 3D culture models.

A microsecond-pulsed dielectric barrier discharge (DBD) system was used to generate NTP directly onto U87 and SC263 spheroids for treatment. Spheroids were then collected immediately (Figure 1B) or 24 h (Figure 1C) after treatment for CD47 analysis. Immunofluorescence staining of spheroid sections revealed that following NTP treatment, spheroids of both cancer types showed modulation of CD47 compared to untreated. This was further confirmed via quantification of fluorescence intensity and normalization to the untreated signal. Compared to untreated, CD47 signal intensity was reduced immediately after NTP treatment for both U87 spheroids (0.86 ± 0.05 vs. 1.00 ± 0.03; *p* ≤ 0.05; Figure 1D) and SC263 spheroids (0.46 ± 0.04 vs. 1.00 ± 0.05; *p* ≤ 0.001; Figure 1E). Analysis at 24 h post treatment showed that CD47 expression returned to baseline untreated levels (Figure 1F,G).

As A375 melanoma cells did not form spheroids, 3D tumors were developed on fertilized avian eggs, also known as an in ovo tumor model, using the chick chorioallantoic membrane (CAM) assay to further investigate the effects of NTP on CD47. Although a much more complex and labor-intensive model compared to the spheroid model, the in ovo tumor model has additional advantages, which allow it to better represent the tumor microenvironment [25,26]. Treatment responses in this model are also more clinically representative compared to other in vitro models, while avoiding the use and sacrifice of live animals required of in vivo experiments [25,26].

**Figure 2:**
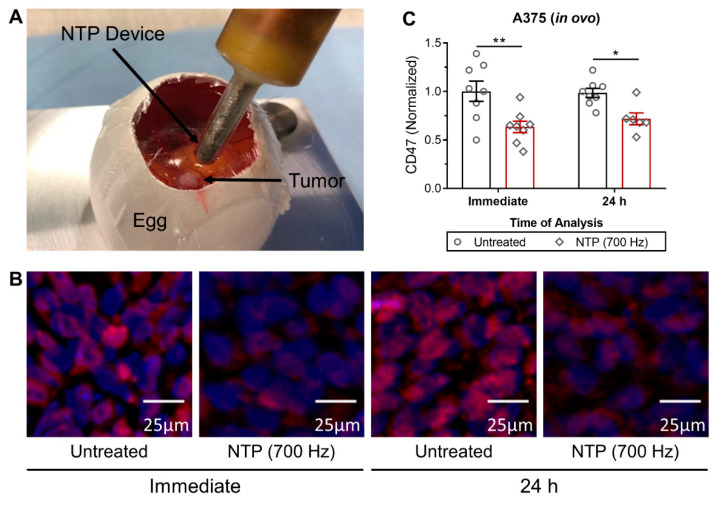
NTP treatment decreased CD47 expression of A375 melanoma tumors in an in ovo model. (**A**) Tumors were grown on the chorioallantoic membrane (CAM) of fertilized eggs and treated directly with NTP. Following treatment, tumors were resected immediately or 24 h post treatment, sectioned, and (**B**) stained for CD47 (red) and counterstained with DAPI (blue). All images were taken on the same day at 10× and batch processed for quantification to limit variations. (**C**) Quantification of CD47 was normalized to the untreated. Data here are represented as mean ± SEM and individual values are also shown (n = 6–8). Statistical significance of all treatment conditions was determined using the generalized linear mixed model with post hoc Dunnett’s test comparison to untreated (* *p* ≤ 0.05; ** *p* ≤ 0.01).

Due to the increased size of the tumor compared to spheroids (Figure 2A), NTP treatment intensity was increased to 700 Hz. Following treatment, CD47 was immediately modulated in the tumor compared to untreated (Figure 2B). Interestingly, the CD47 signal remained below that of the untreated even at 24 h (Figure 2B). This was further confirmed via quantification of the fluorescence intensity in representative areas within the tumor (Figure 2C). Areas comprised only of human cells were chosen, and areas with a high mixture of chicken cells were avoided to prevent non-specific analysis (Figure S2). All slides were stained and imaged on the same day, and all images were batch processed to limit variation. CD47 was modulated immediately after NTP treatment (0.64 ± 0.06; *p* ≤ 0.01) and only recovered slightly at 24 h (0.72 ± 0.06; *p* ≤ 0.05).

### 3.2. NTP Effect on CD47 In Vivo

**Figure 3:**
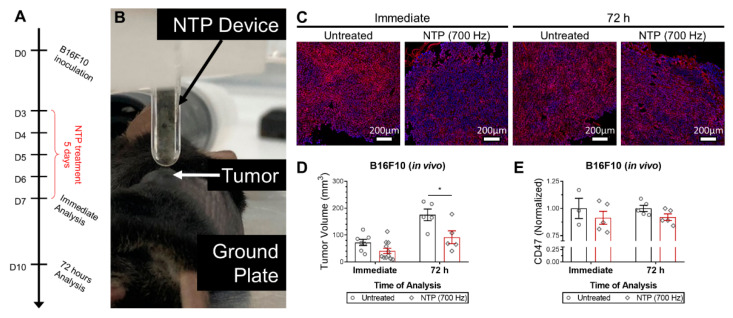
NTP treatment reduced tumor volume and decreased CD47 expression, slightly, in vivo. (**A**) A schematic of the experimental design is provided. (**B**) B16F10 melanoma tumors were established in syngeneic B57BL/6 mice and treated directly with NTP for 5 consecutive days. Following treatment, tumors were resected immediately or 72 h post treatment and (**C**) stained for CD47 (red) and counterstained with DAPI (blue). All images were taken on the same day at 20× and batch processed for quantification to limit variations. (**D**) Tumor volumes were also reduced after treatment. (**E**) Quantification of CD47 was normalized to the untreated. Data here are represented as mean ± SEM and individual values are also shown (n = 3–11). Statistical significance of all treatment conditions was determined using the generalized linear mixed model with post hoc Dunnett’s test comparison to untreated (* *p* ≤ 0.05).

Having demonstrated that NTP reduced CD47 in vitro, we evaluated if the effect would translate in an animal model. Subcutaneous B16F10 melanoma tumors were established in syngeneic C57BL6 mice, and treated with NTP for five consecutive days (Figure 3A,B). At the end of the 5-day treatment, tumor volumes compared to untreated were reduced (41.1 ± 10.1 vs. 72.4 ± 11.6 mm^3^; *p* = 0.064; Figure 3D). By 72 h after the last treatment, the difference between the NTP-treated and untreated tumors was even greater (91.9 ± 24.0 vs. 175.3 ± 21.6 mm^3^; *p* ≤ 0.05; Figure 3D).

Tumors were collected after the last treatment or 72 h afterwards and evaluated for CD47 expression (Figure 3C). We observed a slight decrease in CD47 expression immediately (0.91 ± 0.06), which persisted for 72 h (0.92 ± 0.03), though the data was not statistically significant (Figure 3E). This could be due to the increased biological variability of the in vivo model. Higher treatment intensities may also be required to reach greater CD47 reduction. However, it is of interest that in both the in ovo and in vivo model, NTP treatment appears to have lasting effects on this CD47, though the exact mechanism is not yet clear.

Taken together, our in vitro and in vivo results demonstrated that NTP treatment immediately modulates CD47. It is important to note that due to the timing of analysis, the immediate reduction in CD47 signal is likely due to decreased binding between surface CD47 and the monoclonal antibody used in this experiment (B6H12.2; BD Biosciences, 556046) and not due to downregulation of cellular CD47 production. To test this, we carried out MD simulations to elucidate the underlying NTP mechanisms of action.

### 3.3. In Silico Investigation of Mechanism of Action

**Figure 4:**
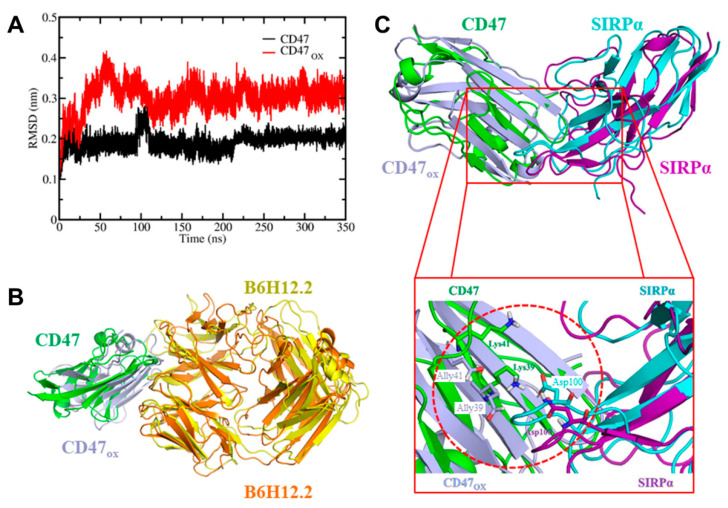
In silico evaluation of the mechanisms of NTP action on immunosuppressive CD47. (**A**) Root mean square deviation (RMSD) of the backbone atoms of the native CD47 and oxidized CD47 (CD47_OX_) structures. (**B**) Cartoon view of aligned native (green and cyan) and oxidized (light blue and purple) CD47–SIRPα systems. Lysine (Lys) is modified into allysine (Ally) after oxidation (Tables S1 and S2). (**C**) Salt bridges are formed between Lys39-Asp100 and Lys41-Asp100 in the native complex and broken after oxidation of CD47 (see circle in inset).

Based on our in vitro observations, we hypothesized that NTP-generated RONS are able to immediately oxidize CD47 and affect its binding affinity to its receptor on immune cells or antibodies. To gain deeper insight into the possible mechanisms of action, we performed MD simulations of a system consisting of native and oxidized CD47 (CD47_OX_). Amino acids involved in the creation of the CD47_OX_ protein are shown in Tables S1 and S2. The oxidation of proteins is known to increase their flexibility, thereby leading to conformational changes in the protein [27,28]. The calculated root mean square deviation (RMSD) of the backbone atoms of the CD47 and CD47_OX_ structures shows that oxidation results in higher fluctuations (Figure 4A). This in turn induces conformational changes in CD47_OX_, and consequently affects its binding affinity. In the in vitro experiments above, surface-exposed CD47 on cancer cells binds with a monoclonal antibody (B6H12.2), whereas in the body, CD47 would bind with SIRPα, an immune receptor found on macrophages, dendritic cells, and other cells of the innate immune system [16]. Considering this, we performed MD simulations for interactions with both the antibody (Figure 4B) and SIRPα (Figure 4C) to gain insight into the experimental and immunologic conditions, respectively. As the results of both cases were similar, the simulation results with the B6H12.2 antibodies are presented in supplementary materials (Figure S4 and Table S3). Simulations revealed that NTP-induced oxidation causes reduced binding of CD47 with its antibody, thus supporting our hypothesis for the modulation of CD47 observed experimentally (Figure 1 and Figure 2). Below, we focus on the simulation results with SIRPα to investigate the potential immunologic consequence of oxidized CD47.

The alignment of the CD47–SIRPα and CD47_OX_–SIRPα systems showed a shift in the loops located at the binding sites of both CD47_OX_ and SIRPα proteins (Figure 4C), which is the consequence of the conformational changes taking place in CD47_OX_. Analysis of the last 50 ns of the MD trajectory reveals that 10 inter-protein salt bridges are formed in the native complex (Table 1), which are located within the cut-off radius used in our simulations (1.4 nm). Of these salt bridges, Lys39-Asp100 and Lys41-Asp100 between CD47 and SIRPα were disrupted after oxidation of the CD47 structure, and the distance between other residues were also altered due to the conformational changes (Table 1 and cf. Figure 4C). The inter-protein salt bridges are known to play a substantial role in intra- and inter-protein interactions and protein conformations [27,29]. Hence, the disruption of these bridges will affect the binding affinity of CD47 to SIRPα. 

**Figure 5:**
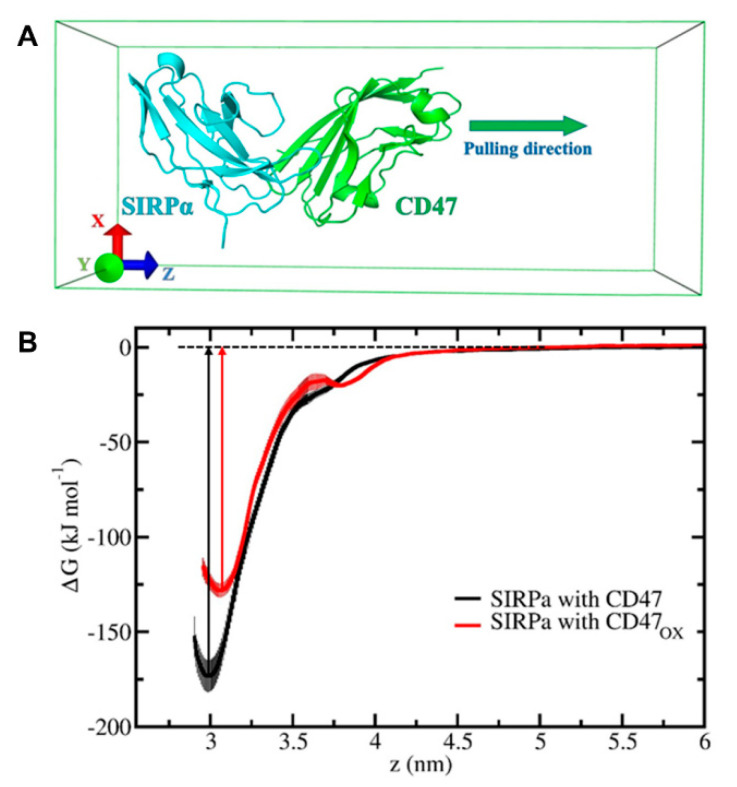
In silico evaluation to determine the effect of CD47 oxidation on binding affinity with SIRPα. (**A**) CD47 and CD47_OX_ were pulled against SIRPα. (**B**) Free energy profiles (FEPs) of the native (black) and oxidized (red) CD47 structures dissociated from SIRPα, indicate the dissociation energy. Errors associated with the sampling are presented in pale color.

To estimate the effect of oxidation on the binding affinity of CD47 with SIRPα, we calculated the free energy profiles (FEPs) employing umbrella sampling (US) simulations. From the FEPs we obtained the dissociation free energies of CD47/CD47_OX_ from SIRPα, based on the difference between the minimum and maximum values. The FEPs reveal that, when CD47 and CD47_OX_ is pulled against SIRPα (Figure 5A), both proteins totally disintegrate from SIRPα already at a distance of 5.0 nm (Figure 5B). The obtained dissociation free energies are −173.4 ± 8.1 and −128.2 ± 4.3 kJ/mol for native and oxidized CD47, respectively. Hence, the free energy of dissociation decreases by ~45 kJ/mol after oxidation, indicating that oxidation of CD47 leads to a weaker binding affinity with SIRPα. From these computational results, we can conclude that binding of CD47 to SIRPα becomes less favorable after oxidation takes place.

## 4. Discussion

In this paper, we investigated the effect of NTP on the innate immunosuppressive checkpoint ligand, CD47, and used in silico techniques to gain insight into the mechanisms of action and immunologic consequence. We provided new fundamental insight into the chemical interactions of NTP and cancer cells, though the clinical effectiveness of these observations are a part of our ongoing investigation.

When NTP is generated, a unique mixture of highly reactive RONS is produced. After exposure to NTP, the CD47 expression on the surface of cancer cells was immediately reduced in 3D in vitro tumor models (Figure 1 and Figure 2). Furthermore, in vivo treatment of melanoma tumors not only reduced tumor burden but also modulated CD47 expression (Figure 3). Due to the timing of the analysis and the rapid reduction of CD47 expression, it is likely that the RONS generated by NTP were oxidizing the protein and affecting its binding affinity. This was confirmed in silico, via RMSD analysis (Figure 4A) and by calculating the FEP of CD47/CD47_OX_ with the monoclonal antibody used for fluorescence staining, B6H12.2 (Figure S4C). CD47 is only one of many immune checkpoints overexpressed on the surface of cancer cells; others include Galectin-9 and programmed cell death ligand 1 (PD-L1) [30]. To gain deeper insight into this, mass spectrometry analysis of these proteins before and after NTP treatment could be employed. NTP-generated RONS could consequently also oxidize these surface markers and affect binding to their immune receptors. Further investigation into the degree of oxidation and the duration before the protein is restored is still required for clinical application.

To investigate the immunologic outcome of NTP-oxidized CD47, we performed MD/US simulations to understand the nanoscale mechanisms of NTP action on the CD47 protein and the subsequent effect on binding affinity with its immune receptor, SIRPα (Figure 4). Computer simulations have played a vital role in studying protein-protein interactions in many different fields [31]. Particularly, simulations help to understand the nature of these interactions at atomic level precision, which is beyond the resolution of an experimental technique. To date, the interaction between cancer cell and immune cell proteins (e.g., CD47–SIRPα) under oxidative stress has not yet been investigated by computer simulations. Our simulation results revealed in detail the disruption mechanism of the binding of CD47_OX_ to SIRPα and show that the free energy of dissociation decreased by ~45 kJ/mol after oxidation (Figure 5B). This suggests that NTP treatment of the tumor can reduce the ability of CD47 to bind to SIRPα on innate immune cells. This would consequently help increase tumor immunogenicity and support the patient’s anti-cancer immune response. Such computer simulations, despite their computational cost, are highly valuable for gaining insights in cancer immunotherapy, without the high financial and labor costs of experimental studies (e.g., autologous/allogenic co-culture assays, in vivo experiments, gene knockout studies).

The results presented here not only add to fundamental knowledge of NTP-cell interactions, but also complement the current understanding that NTP can increase tumor immunogenicity via induction of immunogenic cancer cell death [8,10,11]. Our lab has previously identified an ICD-inducing regime of NTP and reported the increase of surface CRT, a pro-phagocytic signal [8]. Furthermore, we demonstrated that the short-lived RONS produced during treatment, were the major effectors in that regime [8]. In the present study, we showed that NTP operated at the same ICD-inducing regime can also increase tumor immunogenicity by reducing the immunosuppressive signal, CD47 (Figure 1 and Figure 2). Interestingly, while CRT was increased at 24 h, we observed here that CD47 was reduced immediately and returned to baseline 24 h later. This suggests that while RONS were required to stimulate cellular pathways to increased CRT expression, CD47 destruction was inflicted via direct oxidation. As mentioned above, the oxidation of CD47 with NTP shown here, provokes intrigue into how NTP can affect other highly expressed immunosuppressive markers and checkpoints. This would open up a new paradigm in ‘plasma oncology’, where NTP could play a role in reducing the strategies used by cancer cells to evade and suppress the immune system.

Taken together, our results suggest a dual role of NTP therapy to simultaneously increase immunogenic signals and reduce immunosuppressive ones. Due to the short lifetimes and reactivity of this unique mixture of RONS, a highly localized treatment can be performed. This could be an advantage over current ICD inducers which are normally systemic or deeply penetrating (e.g., anthracycline chemotherapeutics, low dose radiation), at least for tumors that are accessible with the NTP device, such as melanoma and head and neck cancer. It remains open to be discovered whether combining NTP with currently available anti-CD47 therapies (e.g., Hu5F9-G4, a humanized IgG4 isotype immune checkpoint inhibitor of CD47) [32,33] will be beneficial or if combination strategies with other checkpoint inhibitors that target a separate axis of anti-cancer immunity are more favorable. Research into NTP for cancer therapy, having only just started in the past decade, has already gained significant momentum, and clinical pilot studies have also been initiated. Metelmann, et al., used NTP to treat patients with advanced head and neck cancer and infected ulcerations [34,35] and Friedman, et al., used NTP to treat actinic keratosis, a common pre-cancerous skin disease [36]. Both studies demonstrated that treatment led to positive responses without short- or long-term adverse effects. It is possible that clinical application of NTP could also be affecting immune responses, although this has not yet been observed or analyzed directly in patients. Studies into both roles of NTP therapy, as we propose, would be of great interest for fundamental understanding and clinical translation.

## 5. Conclusions

In summary, we demonstrate that NTP is able to modulate the immune checkpoint CD47 in 3D cancer spheroids and tumors. IHC analysis showed that all 3 human cell lines of glioblastoma, melanoma, and head and neck squamous cell carcinoma had reduced CD47 expression immediately after NTP treatment compared to controls. Due to the timing of treatment and analysis, this was likely due to oxidation of the protein and was further studied in silico. In silico evaluation revealed higher fluctuations in the RMSD of the backbone atoms of oxidized CD47 compared to the native, which indicate conformational changes to the protein. MD simulations with CD47 and its receptor SIRPα revealed 10 inter-protein salt bridges that were modified following oxidation of CD47, with Lys39-Asp100 and Lys41-Asp100 being completely disrupted. This lead to weaker binding affinity to SIRPα as shown via US simulations and the obtained FEPs. Taken together, our results suggest a role of non-thermal plasma therapy to reduce immunosuppressive signals on tumor cells. The clinical impact of this paradigm requires further investigation.

## Figures and Tables

**Table 1:** Calculated salt bridge distances formed between CD47/CD47_OX_ and SIRPα systems.

No.	CD47/CD47_OX_	SIRPα	N-O Distance Forming Salt Bridge (Å)	
			Native	Oxidized
1	Glu29	Arg69	6.53 ± 1.74	7.60 ± 0.76
2	Glu35	Arg69	5.37 ± 3.37	2.43 ± 0.33
3	Lys39/Ally39	Asp100	3.55 ± 0.82	—
4	Lys41/Ally41	Asp100	7.39 ± 1.05	—
5	Asp51	Arg95	7.24 ± 1.97	7.64 ± 1.06
6	Glu97	Lys53	5.64 ± 1.57	4.74 ± 167
7	Glu97	Lys96	3.88 ± 0.95	4.89 ± 1.33
8	Glu104	Lys53	5.71 ± 1.29	4.82 ± 1.20
9	Glu106	Lys53	3.66 ± 0.74	4.31 ± 1.03
10	Glu106	Lys96	8.13 ± 0.91	8.38 ± 1.34

## Data Availability

The data presented in this study are available on request from the corresponding author.

## Supplementary Materials

The following are available online at https://www.mdpi.com/2072-6694/13/3/579/s1, Figure S1: Gating strategy of untreated A375 cells as a representation of flow cytometry analysis of all cell lines, Figure S2: Representative image of an A375 tumor section resected from the in ovo model, Figure S3: A375 melanoma cancer cells failed to form tumor spheroids, Figure S4: In silico evaluation of the mechanisms of NTP action on CD47 and B6H12.2, Table S1: Amino acids (AA) involved in the creation of the oxidized CD47 protein, Table S2: Chemical structures of original and modified AAs in CD47/CD47OX proteins, Table S3: Calculated salt bridge distances formed between CD47/CD47OX and B6H12.2 systems.
