# Supplementary material for: Oxidation of Innate Immune Checkpoint CD47 on Cancer Cells with Non-Thermal Plasma
Source: Cancers (Basel). 2021 Feb 2;13(3):579. doi: 10.3390/cancers13030579 (PMC7867277; doi:10.3390/cancers13030579)
Supplement: Supplementary file 1 [file cancers-13-00579-s001.pdf]

cancers

Supplementary Materials

Oxidation of Innate Immune Checkpoint CD47 on Cancer Cells with Non-Thermal Plasma

Abraham Lin, Jamoliddin Razzokov, Hanne Verswyvel, Angela Privat-Maldonado, Joey De Backer and Maksudbek Yusupov

1. Gating Strategy for CD47

The glioblastoma (U87), melanoma (A375), and head and neck cancer squamous cell carcinoma (SC263) cell lines were dual stained with an anti-CD47 monoclonal antibody and a live-dead stain (7AAD). For flow cytometry analysis, cells were first gated on the singlets based on forward scatter height (FSC-H) and forward scatter area (FSC-A), and then gated on morphology based on FSC-A and side scatter area (SCC-A). Within this population, cells were then gated on the live population, based on the 7AAD staining (7AAD^{-} population). CD47 analysis was only performed on the live cell population to avoid non-specific staining of dead/permeabilized cells. The gating strategy on untreated A375 cells is shown in figure S1.

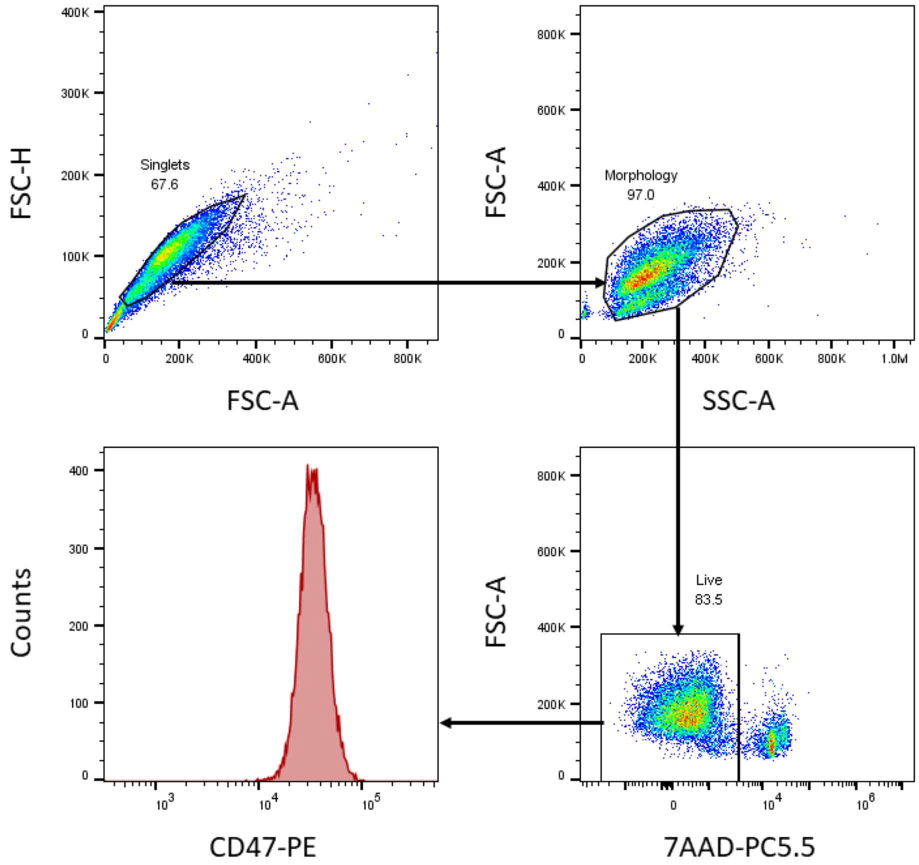

Figure S1. Gating strategy of untreated A375 cells as a representation of flow cytometry analysis of all cell lines.

2. Regions of Interest for A375 *In Ovo* Analysis

Since A375 could not be used to make 3D spheroids, the *in ovo* model was used to develop melanoma tumors. Tumors were grown on the chorioallantoic membrane (CAM) of live chicken embryos and resected at the end of the experiment for immunohistochemical evaluation of CD47. Based on cell size and density, representative sections with only human cells were chosen for analysis of CD47 (figure S2).

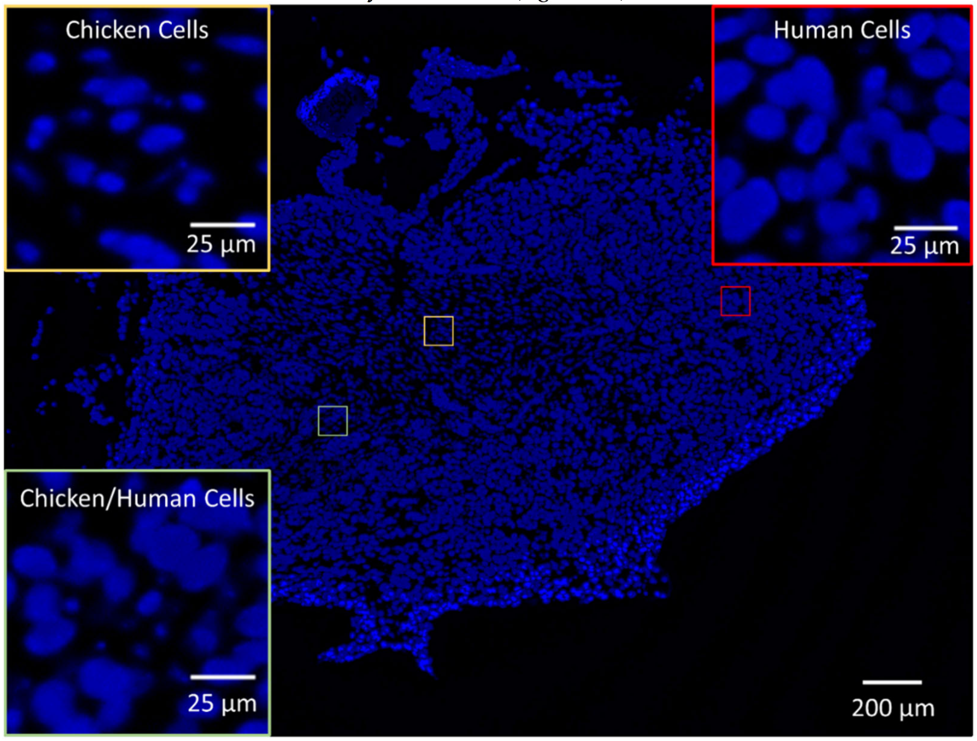

Figure S2. Representative image of an A375 tumor section resected from the *in ovo* model. The nuclei were stained with DAPI (blue). Chicken cells (yellow insert) are generally smaller and less dense compared to human cells (red insert). Areas with both chicken/human cells (green insert) were not chosen for representative analysis of CD47 to avoid non-specific measurements.

3. Making 3D Spheroids with A375 Melanoma Cancer Cells

We attempted to make 3D spheroids with A375 melanoma cancer cells. Even with the use of different supportive matrices, delineated spheroids could not be obtained (figure S3).

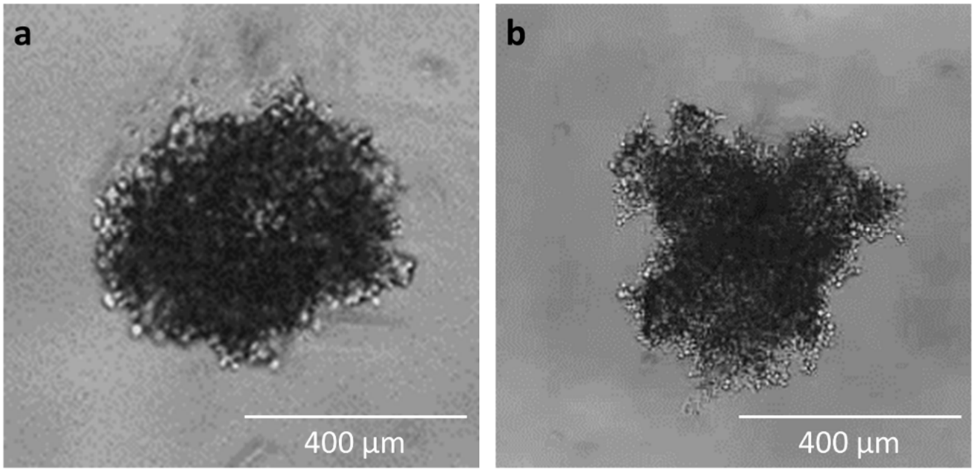

Figure S3. A375 melanoma cancer cells failed to form tumor spheroids. Cells were seeded at a concentration of 5000 cells/well and with a supportive matrix: (**A**) 2% Matrigel or (**B**) 0.24% methocel: Pictures were taken with the EVOS® microscope. Scale bar indicates 400µm.

4. Creation of the Oxidized CD47 Protein for the *In Silico* Study

We selected specific residues in CD47 for oxidation based on our computational results of the solvent accessible surface area (SASA) of the various amino acids (AAs) and the experimental observations of Takai et al. (5), assuming high probability of their interaction with plasma-generated RONS. The selected AAs for oxidation are given in Table S1, and the schematic representations of the original and modified AAs are illustrated in Table S2. 17% of the residues were oxidized to create the oxidized structure of CD47, named CD47ox. CD47ox was prepared by means of the Viena-PTM 2.0 (6) web server, through substituting the residues of the native CD47 with the oxidized ones. The GROMOS45a3 force field parameters of the oxidized residues were obtained from Petrov et al. (7).

Table S1. Amino acids (AA) involved in the creation of the oxidized CD47 protein.

AA in native CD47	*modified AA in CD47ox*
methionine (Met) – 28	methionine sulfoxide
phenylalanine (Phe) –14, 24	3,4-dihydroxyphenylalanine
lysine (Lys) – 6, 39, 41, 43, 56, 67, 75, 84, 112	allysine
tyrosine (Tyr) – 37	3,4-dihydroxyphenylalanine
valine (Val) – 20, 59, 70, 88, 115	3-hydroxyvaline
leucine (Leu) – 3, 54	4-hydroxyleucine

Table S2. Chemical structures of original and modified AAs in CD47/CD47OX proteins.

AA in native CD47	***modified AA in CD47$_{OX}$***
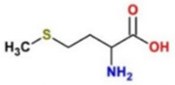 methionine (Met)	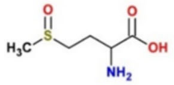 methionine sulfoxide
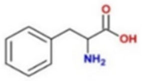 phenylalanine (Phe)	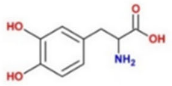 3,4-dihydroxyphenylalanine
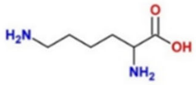 lysine (Lys)	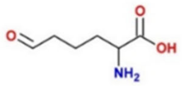 allysine
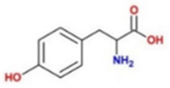 tyrosine (Tyr)	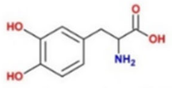 3,4-dihydroxyphenylalanine
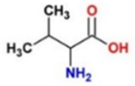 valine (Val)	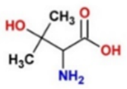 3-hydroxyvaline
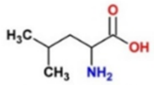 leucine (Leu)	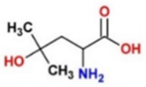 4-hydroxyleucine

4. Molecular Dynamic (MD) Simulations of CD47–B6H12.2

The structures of the CD47–B6H12.2 complex protein system used in our simulations are shown in figure S4A. The aligned structures of both equilibrated CD47–B6H12.2 and CD47$_{OX}$–B6H12.2 complexes are shown in figure S4B. The free energy profile of both the native and oxidized CD47–B6H12.2 system was calculated (figure S4C). The CD47–B6H12.2 complex structure was positioned in the center of the triclinic box. The size of the simulation box was chosen so that it was at a distance of at least 1.1 nm from the atoms at the boundary of the complex system. Consequently, the box was solvated with the SPC water model (8) surrounding the CD47–B6H12.2 complex and 0.1 M of NaCl was added to neutralize the charge of the system. The calculated distances between the AAs of CD47/CD47$_{OX}$ and B6H12.2 that form salt bridges, are given in Table S3.

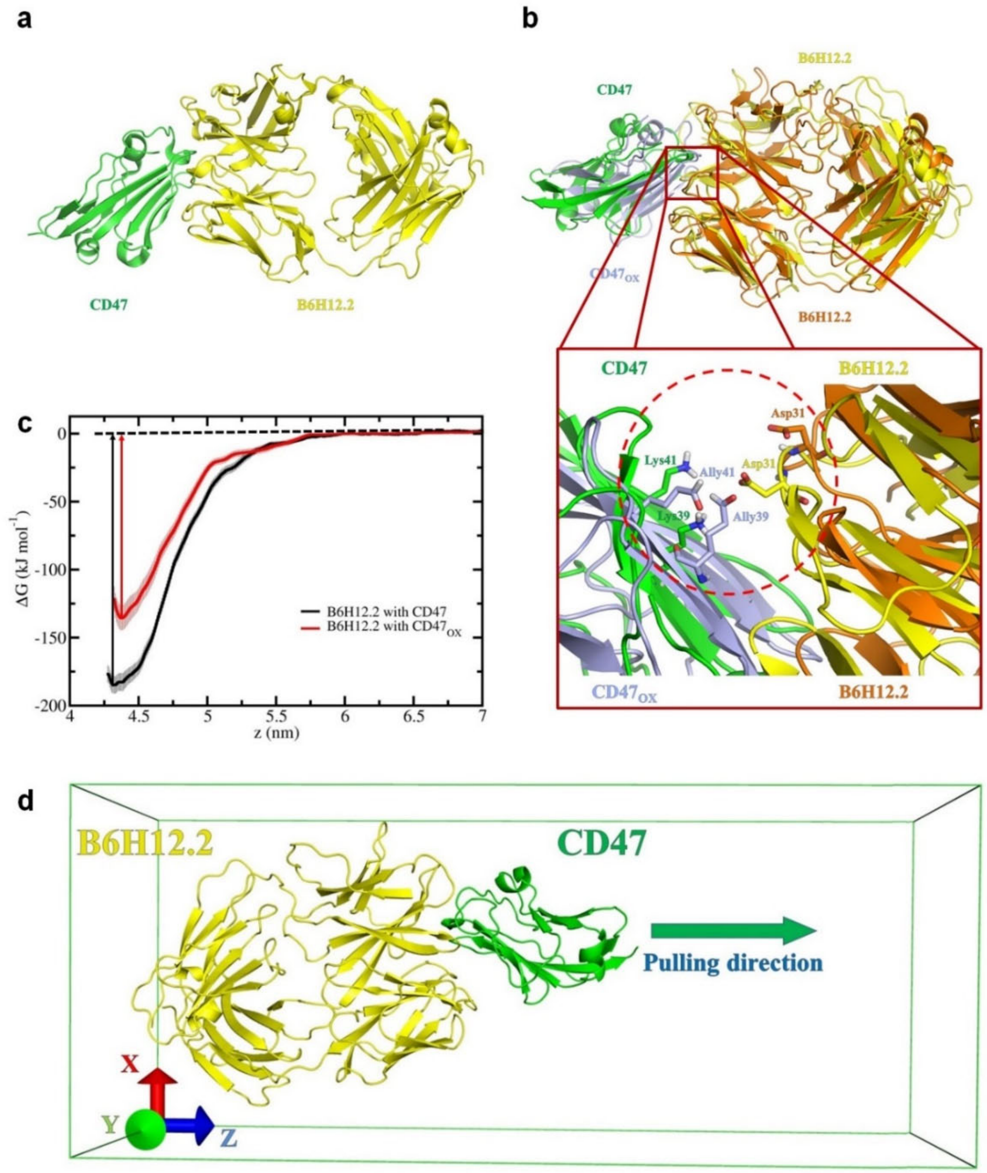

Figure S4. In silico evaluation of the mechanisms of NTP action on CD47 and B6H12.2. (**A**) Cartoon view of CD47 (green) and B6H12.2 (yellow) complex protein system. (**B**) Cartoon view of the aligned structures of CD47–B6H12.2 (green and yellow) and CD47OX–B6H12.2 (light blue and orange) complexes. Highlighted is the cartoon view of aligned native and oxidized systems. Salt bridges are formed between Lys39-Asp31 and Lys41-Asp31 in the native complex. These bridges are broken after oxidation of CD47. (**C**) Free energy profiles (FEPs) of the native (black) and oxidized (red) CD47 structures dissociated from B6H12.2. The errors associated with sampling are presented in pale color. (**D**) The simulation box used in the pulling and US simulations.

Table S3. Calculated salt bridge distances formed between CD47/CD47ox and B6H12.2 systems.

	CD47/CD47ox	B6H12.2	N-O distance forming salt bridge (Å)	
			Native	Oxidized
1	Lys39/Ally39	Asp31	4.64±1.10	-
2	Lys41/Ally41	Asp31	4.99±0.97	-
3	Asp51	Lys49	6.75±1.63	4.67±1.31
4	Glu97	His92	4.87±0.93	5.33±1.24
5	Glu104	His92	6.01±1.05	5.90±0.84
6	Glu104	Arg96	5.93±1.91	4.09±0.80

References

1. Lin, A. G.; Xiang, B.; Merlino, D. J.; Baybutt, T. R.; Sahu, J.; Fridman, A.; Snook, A. E.; Miller, V., Non-thermal plasma induces immunogenic cell death in vivo in murine CT26 colorectal tumors. *Oncoimmunology* **2018**, 1-13.
2. Ivanov, D. P.; Grabowska, A. M., Spheroid arrays for high-throughput single-cell analysis of spatial patterns and biomarker expression in 3D. *Sci. Rep.* **2017,** *7* (1), 1-12.
3. Schuler, L. D.; Daura, X.; Van Gunsteren, W. F., An improved GROMOS96 force field for aliphatic hydrocarbons in the condensed phase. *Journal of Computational Chemistry* **2001,** *22* (11), 1205-1218.
4. Kumar, S.; Rosenberg, J. M.; Bouzida, D.; Swendsen, R. H.; Kollman, P. A., The weighted histogram analysis method for free-energy calculations on biomolecules. I. The method. *Journal of computational chemistry* **1992,** *13* (8), 1011-1021.
5. Takai, E. *et al.* Chemical modification of amino acids by atmospheric-pressure cold plasma in aqueous solution. *Journal of Physics D: Applied Physics* **47**, 285403 (2014).
6. Margreitter, C., Petrov, D. & Zagrovic, B. Vienna-PTM web server: a toolkit for MD simulations of protein post-translational modifications. *Nucleic acids research* **41**, W422-W426 (2013).
7. Petrov, D., Margreitter, C., Grandits, M., Oostenbrink, C. & Zagrovic, B. A systematic framework for molecular dynamics simulations of protein post-translational modifications. *PLoS computational biology* **9**, e1003154 (2013).
8. Berendsen, H. J., Postma, J. P., van Gunsteren, W. F. & Hermans, J. in *Intermolecular forces* 331-342 (Springer, 1981).
